# Ageism and Self-Perception of Ageing: Psychosocial Predictors of Attitudes Towards Ageing

**DOI:** 10.3390/bs16040527

**Published:** 2026-04-01

**Authors:** José María Faílde Garrido, María Dolores Dapía Conde, Laura Ruiz Soriano, Antía Rivera Nieto

**Affiliations:** 1 Longevity and Aging Research Group, Faculty of Education and Social Work, University of Vigo, 32004 Ourense, Spain; ddapia@uvigo.es (M.D.D.C.); lruiz@uvigo.gal (L.R.S.); antia.rivera.nieto@alumnado.uvigo.gal (A.R.N.); 2 Galician Society of Gerontology and Geriatrics, Colexio Oficial de Médicos de Santiago de Compostela, 15701 Santiago de Compostela, Spain

**Keywords:** ageism, attitudes ageing, successful ageing, self-perception

## Abstract

Ageism—encompassing stereotypes, prejudice, and discrimination across age groups—affects how individuals perceive and experience their own ageing. This study, based on a large sample (N = 1047), compared three age cohorts and explored intra-group variability among older adults (65–75 vs. ≥76 years). Results indicated that attitudes towards ageing were influenced by life stage, knowledge about ageing, perceived ageism, and internalised stereotypes. Participants aged 65–75 years showed more favourable attitudes, greater knowledge, and better emotional wellbeing compared to the ≥76 group, which exhibited higher hostile ageism and lower psychological wellbeing. A forward stepwise logistic regression (explained 35.9% of the variance) identified five predictors of a positive self-perception of ageing: lower perceived age discrimination; generally positive attitudinal profile; endorsement of benevolent stereotypes; absence of hostile ageism; and belonging to the 65–75 group. The findings highlighted the psychosocial complexity of ageing and call for interventions promoting positive ageing and reducing ageism.

## 1. Introduction

Global demographic transformation has been marked by an accelerated ageing of the population, a phenomenon that affects all countries and is projected to increase steadily in the coming decades (United Nations, 2024). In the context of the so-called “longevity society”, characterised by longer lifespans and a growing proportion of older people, the study of ageism has become a priority in gerontological research and international policy-making, as it has been identified as a critical barrier to healthy ageing (WHO, 2021). The Joint Research Centre (JRC, 2023) has described ageism as “A challenge for a society of longevity”, highlighting its structural and cultural nature. Ageism impacts not only the way older people are perceived by society, but also their own self-image. It is estimated that one in two people in the world manifests ageist attitudes towards older adults, making this phenomenon one of the most prevalent and least visible forms of exclusion in contemporary societies (WHO, 2021).

The concept of ageism was introduced by the gerontologist, Robert Butler, in 1969, who defined it as “prejudice by one age group against another age group” (Butler, 1969, p. 243), establishing a parallel with forms of discrimination such as racism or sexism. Later contributions have underscored these distinctive features. Levy (2001) conceptualised ageism as an “enemy within”, referring to a type of discrimination that can be directed towards oneself, operating implicitly and consolidating across the lifespan. Its impact is universal, as all people are potentially exposed to ageist attitudes as they age (Ayalon, 2014). Ayalon and Tesch-Römer (2018) defined ageism as “the complex, often negative construction of old age, which takes place at the individual and the societal levels” (p. 3), emphasising its multidimensional nature, with behavioural, affective, and cognitive components.

Starting from this multidimensional conception, theoretical models have deepened our understanding of how stereotypes associated with old age shape manifestations of ageism. The Stereotype Content Model (Fiske et al., 2002) has proposed that social groups are evaluated according to two universal dimensions: communality (agreeableness) and competence (instrumentality). In the case of older people, an ambivalent stereotype predominates that perceives them as warm but not very competent (Cuddy et al., 2005), giving rise to two forms of ageism: benevolent, with paternalistic attitudes that restrict autonomy (Cary et al., 2017), and hostile, based on rejection or social exclusion (Daniel, 2019). This phenomenon can manifest itself explicitly or implicitly, and in various contexts—health, work, family or media—making it a form of cross-cutting discrimination. It also influences attitudes towards ageing itself, favouring internalised or self-inflicted ageism, with negative effects on self-esteem, health, and life expectancy (Levy et al., 2002; WHO, 2021). Communicative styles such as elderspeak reinforce infantilisation and perceived disability, affecting older adults’ social interaction and self-image (Williams et al., 2004). Perceived discrimination tends to increase with age, with older adults reporting higher levels of perceived discrimination, and considering benevolent ageism more acceptable than hostile ageism (Giasson et al., 2017; Gans et al., 2023).

Numerous studies show that ageism is internalised from the early stages of development as a result of socialisation and cultural learning processes. From the age of three, children already display negative stereotypes about old age, associating it with physical deterioration, dependence, and passivity (Bergman, 2019; Flamion et al., 2020; Guardabassi, 2025; WHO, 2021). The association between ageist attitudes and health in older people has been widely documented. Those individuals who perceive greater ageism report lower levels of life satisfaction, higher prevalence of depression, anxiety and stress, poorer adherence to medical treatments, poorer self-care, and higher risk of cognitive decline (Burnes et al., 2019; Chang et al., 2020; Hajek & König, 2024; Hu et al., 2021; Kang & Kim, 2022; Levy, 2022; Shimizu et al., 2022).

Beyond global attitudes towards older adults, research has also examined subjective ageing, including attitudes towards own ageing (ATOA) as a central dimension of how people appraise their own ageing (Lawton, 1975). ATOA has been linked to physical and mental health, wellbeing, life satisfaction, and cognitive functioning, even after controlling for chronological and subjective age (Yang et al., 2024; Yuan et al., 2024). Recent work has indicated that ATOA can act as a mediating mechanism in the relationship between social support and loneliness or psychological distress, including depression, anxiety, and stress (Song et al., 2024). These findings suggest that individuals’ evaluations of their own ageing process constitute a key outcome in the study of ageism and healthy ageing.

Although the negative impact of ageism on the health of older people has been robustly demonstrated (Levy, 2022; WHO, 2021), there is still a scarcity of studies that describe age-related patterns of ageism and attitudes towards ageing across the adult lifespan, especially using designs that allow a fine-grained analysis of different age cohorts (Fernández-Ballesteros & Sánchez-Izquierdo, 2021; Levy, 2003). Existing evidence points to the internalisation of stereotypes throughout life and suggests that people who do not report experiences of ageism tend to manifest more positive attitudes towards ageing (Bergman & Palgi, 2021; Giasson et al., 2017), reinforcing the need to systematically explore how ageist beliefs, perceived discrimination, and knowledge about ageing relate to attitudes towards one’s own ageing in different age groups. Examining age-related patterns across young, middle-aged, and older adults, as well as within old age (e.g., 65–75 vs. 76+), is therefore particularly meaningful. Rather than treating “older adults” as a homogeneous category, a life course perspective allows us to capture how internalised age stereotypes and self-perceptions of ageing may shift as individuals transition between life stages. This approach also underscores that differences in attitudes towards ageing among middle-aged and older adults are not only statistically detectable but also theoretically and clinically relevant, as they may signal windows of increased vulnerability or opportunity for prevention and intervention. However, cross sectional studies can only delineate descriptive age patterns, and longitudinal research is still needed to understand how these attitudes change over time and which factors shape their development (Fernández-Ballesteros & Sánchez-Izquierdo, 2021; Levy, 2003).

In this context, the present study focuses on attitudes towards one’s own ageing as the main outcome and examines how they vary as a function of chronological age and ageism-related factors. Using cross-sectional data from a large sample of adults living in Galicia (Spain), the study has two objectives: (1) to describe age-related patterns in social representations, knowledge about ageing, and ageist attitudes through a comparative analysis across age groups, adopting both an inter-group perspective (young adults, middle-aged adults, and older adults) and an intra-group perspective within older adults (65–75 vs. ≥76 years); and (2) to determine the predictive power of knowledge about ageing, ageist attitudes, and sociodemographic factors on self-perceptions of ageing in adults aged 65 and over.

By focusing on attitudes towards one’s own ageing as the main outcome and modifiable psychosocial factors such as knowledge about ageing, ageist attitudes, and perceived discrimination, this study goes beyond age as a non-modifiable characteristic and highlights potential leverage points for change. Identifying distinct age group patterns and psychosocial predictors of positive self-perceptions of ageing can inform age-tailored psychoeducational programmes, guide clinical efforts to foster resilience against internalised ageism, and support public policies aimed at promoting healthy and inclusive ageing in longevity societies.

## 2. Method

### 2.1. Participants

A total of 1047 people over 18 years of age participated, with an age range between 18 and 98 years, living in different municipalities of Galicia (Spain)—a highly aged region in Spain—and the mean age was 52.81 (SD = 18.57) with 40.4% men and 59.6% women. They were selected by cluster sampling with quotas by age and gender, representative of the adult Galician population. Subsequently, municipalities were selected according to habitat typology (rural, semi-urban, urban).

For the purposes of empirical testing, the groups were first stratified into three age groups (inter-group perspective): young adults (18–35 years old, 19.9%), middle-aged adults (36–64 years old, 52.2%), and older adults (≥65 years old, 28.9%). The latter group was further subdivided into two (intra-group perspective): adults aged 65–75 years and adults aged 76 years and older.

### 2.2. Instruments 

A structured battery of validated instruments was used for data collection, as described below, as well as a block of socio-demographic questions.

*A Questionnaire of Reasons Why a Person is Considered to Have Entered Old Age* (REV)—this instrument was developed by the *Centro de Investigaciones Sociológicas* (CIS, 2008) and adapted for this study. It allowed us to investigate the social perceptions associated with the onset of the ageing process. Participants were asked to select three out of seven predefined response options that would justify their consideration that a person has entered the stage of old age.

*The Facts on Ageing Quiz-1* (Palmore, 1998) consisted of 25 multiple-choice items related to knowledge about physical, mental, and social aspects of ageing, including its stereotypes. Each item has only one correct answer out of four alternatives. The total score ranged from 0 to 25, reflecting a higher level of knowledge as the score increased. In addition, the analysis of the responses allowed the calculation of three additional measures of positive bias, negative bias, and net attitudinal tendency, according to the number of favourable, unfavourable, or neutral choices towards old age. The Facts on Ageing Quiz-1 showed only a moderate internal consistency and construct validity (Palmore, 1998).

*An Ambivalent Ageism Scale* (AAE; Cary et al., 2017)—this instrument assessed the hostile and benevolent components of ageist attitudes. It consisted of 13 items with a Likert-type response format of 1 to 7 points ranging from 1 (strongly disagree) to 7 (strongly agree). It allowed scores to be obtained on two components of old-age stereotypes: hostile (four items) and benevolent (nine items). Cronbach’s alpha for the total scale was 0.86, which was also adequate for the benevolent (α = 0.86) and hostile (α = 0.78) components. Moreover, its benevolent and hostile ageism subscales demonstrated construct validity by correlating in the expected direction with attitudes and perceptions towards older adults (Cary et al., 2017).

*The Questionnaire on Discriminatory Behaviours towards Old Age and Ageing* (CONDUC-ENV; Castellano & de Miguel, 2011) assessed perceived age-related discrimination in everyday contexts. It comprised 31 items rated on a four-point scale (from “never” to “always”) across five domains: health, daily living, family interaction, social interaction, and personal sphere. The scale yielded eight first-order factors (e.g., negative personal attention, supportive treatment, age-related criticism, social inconsideration) and two second-order factors: (1) negative age-based discriminatory behaviours and (2) marginalising behaviours. In our study, the overall internal consistency was acceptable (α = 0.78), with satisfactory reliability for factor 1 (α = 0.78) and modest reliability for factor 2 (α = 0.61). The CONDUC-ENV showed good internal reliability and evidence of construct validity, supported by its bifactorial structure and the factor analyses conducted by the authors.

The *Philadelphia Geriatric Center Morale Scale* (Lawton, 1975; Spanish version: Montorio, 1994) measured general psychological wellbeing in older adults. It included 16 dichotomous (yes/no) items, yielding a total score between 0 and 16, with higher scores reflecting greater morale. The instrument comprised three subscales: agitation, attitudes towards own ageing, and dissatisfaction with loneliness. Lawton reported high internal consistency for the subscales (α = 0.81–0.85), supporting its reliability. In our study, the scale demonstrated similarly robust psychometric properties. The Philadelphia Geriatric Center Morale Scale is a classic instrument for assessing psychological wellbeing in older adults, with good internal reliability and consistent evidence of construct validity across different countries and versions (Montorio, 1994).

### 2.3. Procedure

Data collection was carried out by a previously trained team through face-to-face administration in the selected municipalities. The battery of questionnaires was administered in a single session, using a paper format or an assisted interview in cases of difficulty. Compliance with the ethical principles established by the APA and the Declaration of Helsinki was guaranteed, including obtaining informed consent.

### 2.4. Data Analysis

A comprehensive statistical approach involving descriptive and inferential procedures was used to analyse the data. In the descriptive phase, measures of central tendency (mean) and dispersion (standard deviation, average ranges and sums of ranks), as well as frequency distributions and percentages, were calculated to characterise the study variables.

For inferential analysis, appropriate contrast tests were used according to the nature of the variables. For categorical variables, the chi-square statistic (*χ*^2^) was used to assess associations between groups. In the case of continuous variables, the assumption of normality was first checked using the Kolmogorov–Smirnov test. Because the data did not meet the normality criteria required for parametric tests, non-parametric tests were used: the Kruskal–Wallis H test, for comparisons between more than two independent groups, and the Mann–Whitney U test, for comparisons between two independent groups or for post hoc contrasts. The effect sizes for group comparisons were estimated using *Hedges’ g*, which provided an unbiased estimate of the standardised mean difference when sample sizes differed between groups.

To identify predictors of a positive self-perception of ageing among older adults (≥65 years), a binary logistic regression analysis with forward stepwise entry was conducted, including ageism-related measures (FAQ-1, AAS, CONDUC-ENV), age group (65–75 vs. ≥76), gender, education, marital status, caregiving experience, and perceived knowledge of ageing as candidate predictors. An a priori power analysis was conducted using G*Power (version 3.1) to estimate the minimum required sample size for detecting small-to-medium effects (α = 0.05, power = 0.80) for the planned group comparisons and logistic regression analyses; the final sample of 1047 participants exceeded this requirement and was therefore deemed adequate to ensure sufficient statistical power.

All statistical analyses were conducted using IBM SPSS Statistics for Windows, version 29.0, and the effect size calculations were performed using GPower, version 3.1 (Heinrich Heine Universität Düsseldorf, Düsseldorf, Germany). 

## 3. Results

In this section, we first conducted a comparative analysis of social representations and attitudes towards ageing in three different age groups—young adults, middle-aged adults, and older adults—paying special attention to the particularities of the group of older people. Secondly, with the aim of deepening the understanding of attitudes towards ageing (ATOA), we focused on the group of older people (≥65 years), segmenting it into two groups by age ranges.

### 3.1. Inter-Group Analysis: Social Representations and Attitudes Towards Ageing

**Figure 1:**
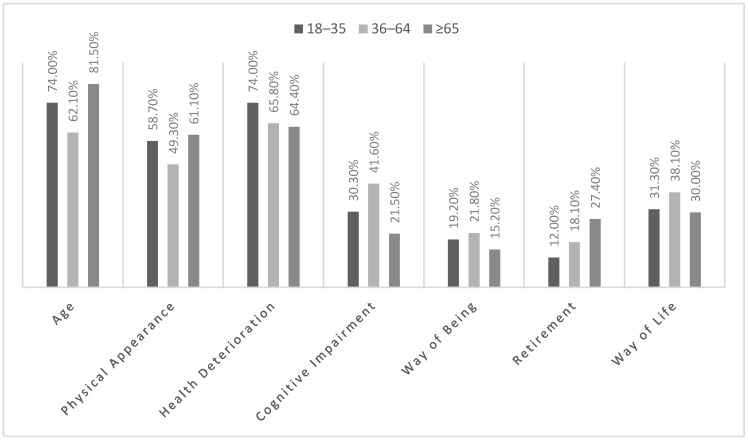
Reasons why a person is considered to have entered old age by age group.

As shown in Figure 1, the most frequent responses in all three groups coincided by pointing to chronological age, physical appearance, and deteriorating health as the main reasons why a person is considered to have entered old age. However, although there was an apparent homogeneity in the criteria for the perception of ageing, there were also relevant nuances depending on the life stage of the participants; the results indicated statistically significant associations in five of the seven reasons chosen to identify older people. Older adults (≥65 years) showed more markedly associated attributes of old age to age (*χ*^2^ = 36.65; *p* > 0.001), physical appearance (*χ*^2^ = 12.65; *p* = 0.002) and being retired (*χ*^2^ = 6.66; *p* = 0.036). In contrast, the middle-aged group associated old age more strongly with intellectual impairment (*χ*^2^ = 36.49; *p* < 0.001) and way of life (*χ*^2^ = 6.66; *p* = 0.036). In particular, the older group tended to value chronological age and physical appearance more highly, while the middle-aged adults referred to elements linked to cognitive functioning and social roles.

The results derived from the *Facts on Ageing Quiz* (FAQ-1) showed insufficient knowledge about ageing in the three age groups assessed (see Table 1); however, there were significant differences in the level of knowledge about ageing between the groups (*χ*^2^ = 14.24; *p* = 0.001). Post hoc tests revealed that the young group scored higher than the middle-aged group (*U* = 44,541.00; *p* < 0.001) and the older group (*U* = 26,171.00; *p* = 0.001), making it the age group with the highest level of factual knowledge about ageing (see Table 1).

In relation to positive bias—understood as distorted beliefs that favoured or idealised older people—significant differences were found between the groups (*χ*^2^ = 50.54; *p* < 0.001); the middle-aged group showed significantly higher scores than the young (*U* = 37,854.00; *p* < 0.001) and old (*U* = 67,023.00; *p* < 0.001) groups, while the older group also scored higher than the young group (*U* = 28,001.50; *p* = 0.029).

Regarding negative bias—understood as pejorative or unfavourable beliefs about older adults—significant differences were also found (*χ*^2^ = 10.31; *p* = 0.006). Ex-post ana-lyses indicated that the middle-aged group had significantly lower scores than the young (*U* = 49,484.00; *p* = 0.017) and older (*U* = 71,885.00; *p* = 0.005) groups, indicating that these two groups held a higher burden of negative biases in relation to old age.

The net attitudinal tendency, obtained from the difference between positive and negative biases, was negative in all three age groups; however, the differences were statistically significant (*χ*^2^ = 32.21; *p* < 0.001). The middle-aged group exhibited a less negative attitude than the young group (*U* = 42,450.50; *p* < 0.001) and the elderly (*U* = 67,468.00; *p* < 0.001), which could denote an intermediate, more ambivalent, or reflective position towards ageing.

Analysis of the Ambivalent Ageism Scale (AAS) results found significant differences between groups in relation to benevolent ageism (*χ*^2^ = 30.95; *p* < 0.001). Older adults (≥65 years) showed significantly lower levels compared to younger age groups (young, *U* = 25,936.00; *p* = 0.001; middle-aged, *U* = 61,897.50; *p* < 0.001).

In hostile ageism, significant differences were detected (*χ*^2^ = 16.66; *p* < 0.001). Post hoc contrasts indicated that the younger group scored significantly lower than the middle-aged (*U* = 44,971.50; *p* < 0.001) and older (*U* = 26,879.50; *p* = 0.004) groups, suggesting a lower presence of explicitly negative attitudes towards older people among the younger participants.

### 3.2. Intra-Group Analysis: Attitudes Towards Ageing in Older People

Disaggregating the group of older adults (aged 65–75 years and 76 years and older) allowed us to more precisely explore the subjective criteria that shaped perceptions of the onset of old age. The three indicators most frequently mentioned as signs of entering old age were chronological age, physical appearance, and deteriorating health, reflecting a conceptualisation centred on biological and aesthetic markers.

However, the inferential analysis showed a statistically significant association in the item referring to age (*χ*^2^ = 7.98, *p* < 0.005), with the *old-old* group presenting a higher percentage of identification with this reason, which could be interpreted as a greater internalisation or self-awareness of the ageing process in the later stages of life.

As can be seen in Table 2, the analysis of the FAQ-1 questionnaire revealed low levels of knowledge about ageing in both age groups (65–75 years and ≥76 years); however, the *young-old* group showed a significantly higher level of knowledge compared to the *old-old* group (*U* = 9565.00, *p* < 0.050; *g* = 0.24).

Regarding attitudinal biases, the negative bias was significantly higher in the *old-old* group (*U* = 9058.50, *p* < 0.050; g = −0.35), maintaining itself in the net attitudinal tendency towards ageism, though this was more unfavourable among older participants (*U* = 9276.50, *p* < 0.005; g = 0.35).

On the other hand, the perceptions from the Questionnaire on Discriminatory Behaviours towards Old Age and Ageing (CONDUC-ENV) found that the old-old group more frequently reported ageist behaviours in their close environment, scoring higher in age-related criticism (*U* = 8381.00, *p* = 0.000; *g* = −0.34); in perceptions of negative treatment due to derogatory attitudes (*U* = 9775.50, *p* < 0.0510; *g* = −0.19); negative family treatment (*U* = 8641.50, *p* < 0.001; *g* = −0.45); and equal treatment (*U* = 9430.00, *p* = 0.050; *g* = −0.28). In contrast, the young-old group reported higher levels of general supportive treatment (*U*= 9636.50, *p* = 0.025; *g* = 0.294) and negative treatment due to social inconsideration (*U* = 9828.00, *p* < 0.050; *g* = 0.26), suggesting a possible differential awareness of the community and family environments.

In the second-order factors (discriminatory behaviours and ageism), no significant differences were observed between the two groups (*p* > 0.05).

Analysis of the *Philadelphia Geriatric Center Morale Scale* (PGMS) showed clear differences between the two groups. As can be seen in Table 3, those in the *young-old* group had significantly higher levels of general psychological wellbeing (*U* = 7374.50, *p* < 0.001; *g* = 0.61), as well as a more positive attitude towards their own ageing (*U* = 7477.50, *p* < 0.001; *g* = 0.60). In contrast, the *old-old* group exhibited higher levels of emotional distress, with higher scores on agitation-anxiety (*U* = 9320.50, *p* = 0.005; *g* = −0.35) and loneliness dissatisfaction (*U* = 7646.00, *p* < 0.001; *g* = −0.59), possibly related to isolation and perceived functional loss.

Finally, a logistic regression analysis was performed to identify predictors of a positive attitude towards one’s own ageing process. The final model, fitted using the forward stepwise selection procedure, identified reliable predictors for the likelihood of adopting a positive attitude towards personal ageing (see Table 4).

The final model retained five significant variables and presented an adequate fit to the data. Specifically, a −2 *log-likelihood* value of 309.629 was obtained, and a *Nagelkerke’s pseudo-R*^2^ of 0.359, which was considered a good level of explanation in psychosocial contexts. Furthermore, the *Hosmer and Lemeshow* goodness-of-fit test was found to be non-significant (*χ*^2^ = 12.155; *gl* = 8; *p* = 0.144), confirming that the model fitted the observed data correctly. Regression analysis showed that negative age-based discriminatory behaviours (β = −0.596, *p* < 0.046); net attitudinal tendency (β = 0.126; *p* = 0.003); benevolent ageism (β = 0.566; *p* < 0.001); hostile ageism (β = −0.948; *p* < 0.001); and age (β = −1.211; *p* < 0.001) significantly predicted the perception of one’s own ageing. The relationship with these variables was negative, except for the benevolent component of ageism and for age; thus, positive attitudes towards ageing were associated with a lower presence of age-related experiences of discrimination, a favourable attitudinal tendency towards ageing, and positive stereotypes towards older people, as well as a lower presence of negative stereotypes and being younger than 76 years old.

Taken together, these results underlined the relevance of attitudinal, social, and generational factors in shaping perceptions of ageing itself, and reinforced the need for interventions tailored to different age groups within old age.

## 4. Discussion

The present study examined attitudes towards one’s own ageing as the central outcome and analysed how these attitudes were related to knowledge about ageing, different manifestations of ageism, and sociodemographic factors in a large adult sample from Spain. Building on previous evidence linking self-perceptions of ageing with physical and mental health, wellbeing, and cognitive functioning, our findings showed that older adults with more favourable attitudes towards their own ageing tended to report lower perceived age discrimination, a more positive attitudinal profile towards old age, and lower levels of hostile ageism, as well as belonging more frequently to the 65–75 age group.

In addition to this predictive focus, we described descriptive age-related patterns in social representations, knowledge about ageing, and ageist attitudes across three age groups (young, middle-aged, and older adults) and among the old age group (65–75 vs. ≥76 years). The intergroup analyses indicated that younger, middle-aged, and older adults differed in their factual knowledge about ageing, the balance between positive and negative age-related biases, and levels of benevolent and hostile ageism. The intra-group analyses among older adults further suggested that the “young-old” (65–75 years) generally presented more favourable profiles in terms of knowledge about ageing and attitudinal tendencies than the “old-old” (≥76 years), highlighting the heterogeneity within older age.

The findings of the present study provided further insight into the psychosocial understanding of ageing, showing how social representations, attitudes towards old age, factual knowledge, attitudinal biases, and internalised ageism were configured according to life stage. The recent scientific literature has confirmed that negative perceptions of ageing not only affected psychological wellbeing but were also associated with adverse physical and mental health indicators (Allen et al., 2022; Bužgová et al., 2024).

From an intergenerational perspective, the results showed that while there was high agreement in attributing certain characteristics as diagnostic for categorising a person as older (chronological age, physical appearance, declining health), statistically differentiated associations between age groups were observed in the attribution of other factors such as intellectual decline, retirement or lifestyle. These differences suggested that social representations of old age were modulated by generational positioning and life experience, which was in line with the findings of Chu et al. (2020) in their review on the conceptual development of attitudes towards ageing.

Factual knowledge about ageing was found to be deficient in all three groups (Faílde Garrido et al., 2020), being paradoxically higher in young adults. This finding was worrying, as factual knowledge about ageing can play a protective role in the development of non-ageist attitudes. Although information was a necessary condition for attitudinal transformation, it was not a sufficient factor to promote positive attitudes towards old age by itself (Soria-Andrés et al., 2024). In this framework, it was necessary not just to improve knowledge about ageing and its processes, but also to accompany it with effective strategies such as quality intergenerational contact, which was configured as an element with transformative potential in shaping such attitudes (Langmann & Weßel, 2023; Manzi et al., 2024).

In terms of attitudes, the results obtained reinforced the hypothesis that ageism was pervasive and cross-cutting (WHO, 2021), confirming the presence of self-directed ageism. These ageist attitudes were not stable throughout the life cycle; this study recognised that age-related stereotypes were constructed and internalised over time. The analysis of attitudes revealed a curvilinear pattern, as middle-aged adults presented a less negative profile, characterised by a greater positive bias and less negative bias, which coincided with research that situated this stage as a turning point in the resignification of the ageing process (Hernández Pérez & Nájera Rodas, 2024). For their part, young people exhibited lower levels of hostile ageism, although also less benevolent bias, which could be interpreted as an affective distancing from ageing (Manzi et al., 2024). In contrast, older people simultaneously showed an increase in both benevolent ageism and negative bias, shaping an ambivalent self-perception influenced by internalised stereotypes (Cary et al., 2017; Kisvetrová et al., 2022; Shimizu et al., 2022).

Conversely, the present study contributed to the understanding of how ageism and self-perceptions of ageing were shaped within the group of older adults. The findings confirmed that attitudes towards ageing were not homogeneous in old age, but varied significantly according to age range, experiences of discrimination, internalised stereotypes, and the emotional state of individuals (Manzi et al., 2024; Shimizu et al., 2022).

The criteria attributed to identify the onset of old age remained constant between the groups aged 65–75 and ≥76 years, with the only exception of age. The significant difference found in the variable “age” as a main indicator suggested that self-perception of ageing can become more pronounced over the years (Allen et al., 2022), probably as a result of internalisation processes of chronological stereotypes.

Although both subgroups had low levels of knowledge about ageing, the young-old group showed significantly higher understanding, which could be related to higher gerontological literacy (Bužgová et al., 2024; Chu et al., 2020). This internal difference has evidenced the need to strengthen continuing education strategies aimed at older people, especially in later life. In addition, recurrent contact with situations of frailty—whether at the individual level or through peer observation—could favour the construction of more negative narratives of ageing, a phenomenon that may be enhanced by upward social comparison processes, as suggested by several studies (Manzi et al., 2024; Allen et al., 2022).

Attitudinally, the old-old group manifested a more negative net attitude towards ageing, characterised by higher negative biases and lower levels of life satisfaction. This pattern was consistent with previous findings associating an unfavourable perception of ageing with lower subjective wellbeing and higher anxiety (Kisvetrová et al., 2022; Manzi et al., 2024). In contrast, older people aged 65–75 years showed better emotional indicators and a more positive attitude, which can be interpreted as greater psychosocial resilience in the early stages of old age (Shimizu et al., 2022).

The perception of ageist behaviours revealed a polarisation based on the established chronological segmentation. Thus, while the old-old group reported more experiences of negative family treatment and negative treatment by others’ derogatory attitudes, the young-old group more frequently identified inconsiderate attitudes in social contexts. This divergence may reflect transformations in support networks and socialisation environments with advancing age (Langmann & Weßel, 2023). Recent studies have shown that everyday ageism—even in subtle forms—has harmful effects on the physical and mental health of older adults, and that its reduction should be a priority in public policies and intervention programmes (Allen et al., 2022; Bužgová et al., 2024; Langmann & Weßel, 2023).

Finally, data from the regression analysis supported the association between ageist attitudes and the perception of ageing itself. That is, fewer experiences of age discrimination, a net positive attitudinal tendency, the presence of benevolent stereotypes, the absence of hostile ageism, and belonging to the 65–75 age group were reliable predictors in analysing the self-appraisal of the ageing process, with hostile ageism and older age acting as psychosocial risk factors conditioning self-appraisal.

One of the most counterintuitive results of the model was the positive association between benevolent stereotypes and favourable self-perceptions of ageing. Despite being considered a subtle form of ageism (e.g., paternalism, overprotection), these stereotypes seemed to reinforce a sense of dignity and appreciation of the ageing process (Kisvetrová et al., 2022). In contrast, the presence of hostile components of ageism acted as a strong inhibitor of positive attitudes, confirming the findings of Shimizu et al. (2022), who linked hostile ageism with lower life satisfaction and a more negative self-evaluation of ageing.

Furthermore, the model confirmed the modulating effect of chronological age, as the young-old group (65–75 years) were more likely to hold a positive attitude, possibly due to less cumulative exposure to conditions of vulnerability. This pattern has been documented by Bužgová et al. (2024), who noted that the early stages of old age tended to be characterised by more resilient and constructive perceptions.

Ultimately, the regression model, which explained 35.9% of the total variance, showed that attitudes towards one’s own ageing were shaped by the interaction of cognitive (beliefs), affective (emotional perception), contextual (social experiences), and generational (life stage) factors, in line with integrative approaches to active and resilient ageing (Manzi et al., 2024).

It is important to emphasise that, due to the cross-sectional design of the study, these results have allowed us to delineate descriptive age-related patterns rather than to infer developmental changes or the evolution of ageism and attitudes towards ageing across the life course. Longitudinal research is needed to clarify how ageist beliefs and self-perceptions of ageing can change over time and which factors can shape their trajectories.

Although this study has a relatively large sample and has been conducted in a markedly ageing environment—Galicia, Spain, with 26.6% of the population aged over 65 years old (INE, 2025)—it has some limitations that should be considered. Its cross-sectional design precluded causal inference, and the age-group segmentation (65–75 vs. ≥76) may have masked intra-group variability (Manzi et al., 2024). The reliance on self-reported measures may have introduced bias, suggesting the value of mixed methods (Chu et al., 2020). Implicit ageism was not assessed, limiting comprehensiveness (Shimizu et al., 2022), nor were resilience variables such as social support, self-esteem, or spirituality, which may have influenced ageing perceptions (Allen et al., 2022).

Overall, our findings underlined the relevance of considering both age-group differences and modifiable psychosocial factors when studying attitudes towards one’s own ageing. While the descriptive age-related patterns provided a contextual framework, the identification of specific psychosocial predictors of positive self-perceptions of ageing can point to potential targets for intervention. Future studies should build on these results using longitudinal designs and more parsimonious models that focus on the most relevant dimensions of ageism and subjective ageing.

## 5. Conclusions

The findings have relevant implications for public policies and psychosocial interventions to combat ageism and promote a positive self-perception of ageing. Multigenerational educational campaigns are recommended to raise awareness of the ambivalent effects of benevolent and hostile stereotypes, and to improve knowledge about ageing (Cary et al., 2017; Kisvetrová et al., 2022; Allen et al., 2022). Targeted psychoeducational interventions for adults aged ≥76 should address cognitive, emotional, and contextual factors using participatory approaches, given the link between favourable attitudes and better ageing perceptions (Manzi et al., 2024; Shimizu et al., 2022). At the institutional level, it is essential to detect ageist behaviours in public services and to train personnel in gerontological competencies (Chu et al., 2020). Promoting age-friendly environments and including attitudinal indicators in programme evaluations should also be encouraged. Longitudinal studies will be needed to assess the long-term impact on psychosocial wellbeing (Bužgová et al., 2024).

## Figures and Tables

**Table 1:** Knowledge and attitudes towards ageing according to age groups.

	Age 18–35 Mean (SD)	Age 36–64 Mean (SD)	Age ≥ 65 Mean (SD)	*χ* ^2^	*Sig.*	Post Hoc (*Hedges’ g*)
**FAQ1_Total score**	10.80 (2.40)	10.05 (2.67)	10.10 (2.44)	14.24	0.001	1 > 2 (0.30) 1 > 3 (0.29)
**FAQ1_Positive bias**	2.65 (1.59)	3.66 (1.91)	3.11 (2.02)	50.54	0.000	2 > 1 (0.59) 2 > 3 (0.30) 3 > 1 (0.24)
**FAQ1_Negative bias**	9.60 (2.37)	9.12 (2.75)	9.62 (2.51)	10.31	0.006	2 < 1 (0.19) 2 < 3 (0.19)
**FAQ1_Attitudinal tendency**	−6.95 (3.37)	−5.46 (3.92)	−6.51 (3.78)	32.21	0.000	2 > 1 (0.39) 2 > 3 (0.27)
**AAS_Benevolent ageism**	3.40 (1.28)	3.34 (1.19)	3.82 (1.38)	30.95	0.000	3 > 1 (0.34) 3 > 2 (0.37)
**AAS_Hostile ageism**	2.42 (1.44)	2.84 (1.40)	2.72 (1.36)	16.66	0.000	1 < 2 (0.29) 1 < 3 (0.21)

Note. FAQ-1 = Facts on Ageing Quiz-1; AAS = Ambivalent Ageism Scale; SD = standard deviation; *χ*^2^ = Chi-square; *Sig.* = significance; *g* = *Hedges’ g*.

**Table 2:** Knowledge and attitudes towards ageing by age group (65–75 vs. 76 and older).

	Young-Old Mean (SD)	Old-Old Mean (SD)	Mann–Whitney *U* *Sig.*	*Hedges’ g*
**FAQ1_Total score**	10.36 (2.37)	9.79 (2.49)	9565.00 0.020	0.24
**FAQ1_Positive bias**	3.33 (2.17)	2.85 (1.79)	10,099.50 0.086	0.24
**FAQ1_Negative bias**	9.23 (2.47)	10.09 (2.48)	9058.50 0.002	−0.35
**FAQ1_Attitudinal tendency**	−5.90 (3.98)	−7.24 (3.40)	9276.50 0.005	0.35
**AAS_Benevolent ageism**	3.78 (1.14)	3.87 (1.63)	10,626.50 0.318	−0.06
**AAS_Hostile ageism**	2.65 (1.29)	2.80 (1.44)	10,824.00 0.456	−0.11
**CONDUC-ENV F-**1**: Negative personal attention**	3.33 (2.32)	3.60 (2.29)	10,313.50 0.153	0.12
**CONDUC-ENV F-**2**: General supportive treatment**	6.89 (2.29)	6.17 (2.60)	9636.50 0.025	0.29
**CONDUC-ENV F-**3**: Favourable treatment characterised by understanding and assistance**	3.06 (1.40)	2.98 (1.75)	11,140.50 0.736	0.05
**CONDUC-ENV F-**4**: Age-related criticism**	2.28 (2.03)	2.95 (1.88)	8381.00 0.000	−0.34
**CONDUC-ENV_F-**5**: Negative treatment due to demeaning attitudes from others**	0.82 (1.30)	1.05 (1.16)	9775.50 0.022	−0.19
**CONDUC-ENV_F-**6**: Normalised equal treatment**	1.92 (1.95)	2.49 (2.11)	9430.00 0.012	−0.28
**CONDUC-ENV_F-**7**: Negative treatment within the family**	3.05 (2.45)	4.21 (2.74)	8641.500 0.000	−0.45
**CONDUC-ENV_F-**8**: Negative treatment due to social disregard**	4.70 (1.34)	4.22 (2.12)	9828.00 0.037	0.26
**CONDUC-ENV_F-I: Negative discriminatory behaviours based on age**	21.97 (8.97)	23.58 (9.45)	10,021.50 0.106	−0.17
**CONDUC-ENV** **_F-II: Marginalising behaviours due to age**	3.88 (2.17)	4.04 (2.10)	10,651.50 0.327	−0.07

Note. FAQ-1 = Facts on Ageing Quiz-1; AAS = Ambivalent Ageism Scale; CONDUC-ENV = Questionnaire on Discriminatory Behaviours towards Old Age and Ageing; SD = standard deviation; *U* = Mann–Whitney U; *Sig.* = significance; *g* = *Hedges’ g*.

**Table 3:** Levels of psychological wellbeing by age group (young-old vs. old-old).

	Young-Old Mean (SD)	Old-Old Mean (SD)	*U* *Sig.*	*Hedges’ g*
**PGMS_Agitation/Anxiety**	3.47 (2.21)	4.21 (1.98)	9320.50 0.005	−0.35
**PGMS_ Loneliness Dissatisfaction**	1.16 (1.22)	1.88 (1.20)	7646.00 0.000	−0.59
**PGMS_Attitudes Towards Own Ageing**	2.33 (1.35)	1.52 (1.33)	7477.500 0.000	0.60
**PGMS_Total Score**	8.60 (3.97)	6.21 (3.65)	7374.50 0.000	0.61

Note. PGMS = Philadelphia Geriatric Center Morale Scale.

**Table 4:** Logistic regression model of final stepwise results predicting attitudes towards own ageing.

Predictor Variable	Coefficient (β)	Standard Error	Wald	*p*-Value	Odds Ratio (Exp(β))	95% CI for Exp(β)
CONDUC-ENV FII_I: Negative Age-Based Discriminatory Behaviours	−0.596	0.299	3.974	0.046	0.551	[0.307–0.990]
FAQ-1: Net Attitudinal Tendency	0.126	0.042	8.962	0.003	1.134	[1.044–1.232]
AAS: Benevolent Ageist Component	0.566	0.132	18.320	0.000	1.760	[1.359–2.281]
AAS: Hostile Ageist Component	−0.948	0.142	44.286	0.000	0.388	[0.293–0.512]
Age Group (65–75 vs. 76 and older)	−1.211	0.295	16.859	0.000	0.298	[0.167–0.531]

Note. FAQ-1 = Facts on Ageing Quiz-1; AAS = Ambivalent Ageism Scale; CONDUC-ENV = Questionnaire on Discriminatory Behaviours towards Old Age and Ageing; CI = confidence interval.

## Data Availability

The data that support the findings of this study are available from the corresponding author upon reasonable request and/or will be deposited in the repository indicated by the journal.
